# Comparative transcriptomics suggest unique molecular adaptations within tardigrade lineages

**DOI:** 10.1186/s12864-019-5912-x

**Published:** 2019-07-24

**Authors:** Maria Kamilari, Aslak Jørgensen, Morten Schiøtt, Nadja Møbjerg

**Affiliations:** 1 Section for Cell Biology and Physiology, Department of Biology, August Krogh Building, University of Copenhagen, Universitetsparken 13, Copenhagen, Denmark; 2 Section for Ecology and Evolution, Department of Biology, University of Copenhagen, Universitetsparken 15, Copenhagen, Denmark

**Keywords:** Cold shock domain, Functional gene categories, Model organisms, Stress genes, Tardigrada, Transcriptomics

## Abstract

**Background:**

Tardigrades are renowned for their ability to enter cryptobiosis (latent life) and endure extreme stress, including desiccation and freezing. Increased focus is on revealing molecular mechanisms underlying this tolerance. Here, we provide the first transcriptomes from the heterotardigrade *Echiniscoides* cf. *sigismundi* and the eutardigrade *Richtersius* cf. *coronifer*, and compare these with data from other tardigrades and six eukaryote models. Investigating 107 genes/gene families, our study provides a thorough analysis of tardigrade gene content with focus on stress tolerance.

**Results:**

*E.* cf. *sigismundi*, a strong cryptobiont, apparently lacks expression of a number of stress related genes. Most conspicuous is the lack of transcripts from genes involved in classical Non-Homologous End Joining. Our analyses suggest that post-cryptobiotic survival in tardigrades could rely on high fidelity transcription-coupled DNA repair. Tardigrades seem to lack many peroxins, but they all have a comprehensive number of genes encoding proteins involved in antioxidant defense. The “tardigrade unique proteins” (CAHS, SAHS, MAHS, RvLEAM), seem to be missing in the heterotardigrade lineage, revealing that cryptobiosis in general cannot be attributed solely to these proteins. Our investigation further reveals a unique and highly expressed cold shock domain. We hypothesize that the cold shock protein acts as a RNA-chaperone involved in regulation of translation following freezing.

**Conclusions:**

Our results show common gene family contractions and expansions within stress related gene pathways in tardigrades, but also indicate that evolutionary lineages have a high degree of divergence. Different taxa and lineages may exhibit unique physiological adaptations towards stress conditions involving possible unknown functional homologues and/or novel physiological and biochemical mechanisms. To further substantiate the current results genome assemblies coupled with transcriptome data and experimental investigations are needed from tardigrades belonging to different evolutionary lineages.

**Electronic supplementary material:**

The online version of this article (10.1186/s12864-019-5912-x) contains supplementary material, which is available to authorized users.

## Background

Tardigrades (water bears) are microscopic ecdysozoans found worldwide in a range of extreme environments [1, 2]. These small animals need a film of water to be active—they have adapted to extreme conditions such as desiccation, freezing and severe osmotic stress by evolving a highly resistant, ametabolic state called cryptobiosis. Curiously, this state gives tardigrades the ability to survive conditions that are far more extreme than those imposed by their natural habitats, including e.g. space conditions [3–6].

Extant tardigrades divide into two major evolutionary lineages represented by Eutardigrada and the more diverse Heterotardigrada [7]. Fossils from Siberian limestone [8] and phylogenetic analyses suggest that the split between the two lineages is dated ca. 600–540 mya. [9, 10]. Eutardigrades are by far the most extensively studied and represent the focus of recent genome, transcriptome and proteome studies [11–17]. The first tardigrade genome published on the parachelan eutardigrade *Hypsibius exemplaris* [13] claimed for massive horizontal gene transfer (ca. 17.5%), but this study was very quickly refuted by other genome assemblies on the same species [14]. Recent advances in eutardigrade omics include the discovery of so-called “tardigrade unique” proteins implicated in tardigrade stress tolerance [15–17].

Here, we provide two new tardigrade transcriptomes and a thorough examination and discussion of genes proposed to be involved in tardigrade stress tolerance. Our aim is to provide a more solid starting point and basis for identifying molecular players involved in cryptobiosis. Specifically, we provide the transcriptome of the marine tidal heterotardigrade *Echiniscoides* cf. *sigismundi*, which holds a unique evolutionary position within Tardigrada [18, 19] and in addition, a new transcriptome of a eutardigrade—i.e., the first transcriptome within the eutardigrade family Richtersiidae, providing a more comprehensive picture of the gene content and expression patterns within Eutardigrada. We compare the two transcriptomes with available genome and transcriptome data from the eutardigrades *Ramazzottius* cf. *varieornatus* and *Hypsibius exemplaris* [20] as well as sequence data from six model organisms spanning Eukaryota.

Our detailed analyses of 107 genes/gene families with a putative role in stress tolerance indicate common contractions and expansions within stress related gene pathways in tardigrades, but also indicate that evolutionary lineages have a high degree of divergence. For the first time we report the presence of a highly expressed core animal cold shock domain in tardigrades, which may be involved in RNA chaperoning. While intrinsic disordered LEA proteins appear to be present in all tardigrade species, our analyses also indicate that the “tardigrade unique” genes, *CAHS*, *SAHS*, *MAHS* and the mitochondrial *RvLEAM*, are present only in the eutardigrade lineage and that all, except *CAHS*, seem restricted to the eutardigrade order Parachela. Thus, the cryptobiotic capabilities of tardigrades in general cannot be attributed to these newly identified proteins emphasizing that different tardigrade lineages have evolved unique molecular adaptations with implications for their stress tolerance. We further highlight evidence for commonalities and divergences among tardigrades within DNA repair, antioxidant defense, and various bioprotectants providing a comprehensive picture of the molecular machinery underlying tardigrade stress tolerance.

## Results

### Global comparison of tardigrade transcriptomes and genomes

In the following we bring an analysis of the two new transcriptomes. As outlined in the Method section, transcriptome sequencing and basic bioinformatics analyses were conducted by BGI, Shenzhen. Statistical summaries of the obtained sequences and their annotation are presented in Tables 1 and 2.

**Table 1:** Statistical summary of RNA-Seq on *Echiniscoides* cf. *sigismundi* and *Richtersius* cf. *coronifer* (data from BGI)

	*Echiniscoides* cf. *sigismundi*	*Richtersius* cf. *coronifer*
Total Bases	5,531,945,800	5,761,908,600
Number of raw reads	58,601,018	64,000,000
Number of clean reads	55,319,458	57,619,086
GC%	34.73%	47.40%
Q20	97.54%	96.63%
Number of contigs	55,499	84,106
Mean length of contigs (bp)	450	454
N50_contigs_	1176	1259
Number of Unigenes	31,601	55,053
Mean length of Unigenes	830	1052
N50_Unigenes_	1524	2197
Number of Unigenes > 1 kb	9974	20,319
Number of Unigenes > 3 kb	1068	4589
FPKM_mean /_ (min-max)	27.3587/(0.0–44,747.82)	13.4950/(0.0–14,668.10)
FPKM_median_	4.6156	2.1674
FPKM_sum_	864,563.2158	742,939.1452
TPM_mean_ / (min-max)	31.6446 (0–51,757.72)	18.1643 (0–19,743.35)
TPM_median_	5.3387	2.9173
Number of Unigenes after filtering^a^	15,784	21,384
Mean length of Unigenes after filtering	1175	1160

^a^For the detailed analysis of stress related genes (see Table 3), we applied the following filtering parameters: FPKM > 1; transcript length > 300 bp; longest contig for each locus (see text for details)

**Table 2:** Annotation results for the *Echiniscoides* cf. *sigismundi* and *Richtersius* cf. *coronifer* transcriptomes (data from BGI)

Annotation	*Echiniscoides* cf. *sigismundi*	*Richtersius* cf. *coronifer*
Number of Unigenes	31,601	55,053
Unigenes with hits in NR database	13,388	20,001
Unigenes with hits in NT database	4334	3865
Unigenes with hits in Swiss-Prot database	12,295	18,132
Unigenes with KEGG pathways	10,519	15,898
Unigenes with hits in COG database	6042	9195
Unigenes with GO terms	6047	10,853
Total annotated Unigenes	14,159	20,326
Protein coding region prediction		
Unigenes mapped to protein databases^a^	13,578	20,043
Unigenes with predicted CDS (ESTscan)	7359	3322

^a^NR, Swiss-Prot, KEGG and COG databases

**Fig. 1:**
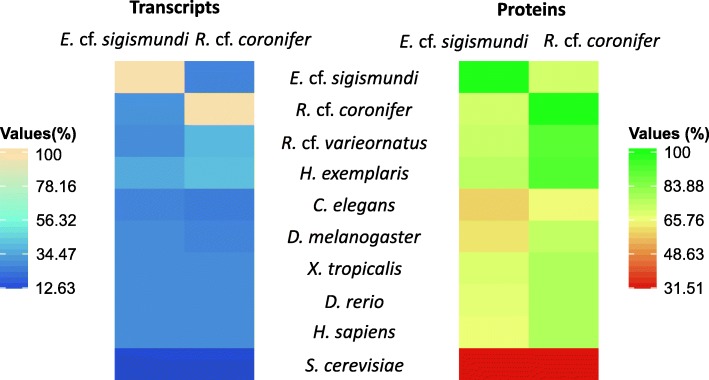
Global comparisons of transcripts and predicted protein sequences. Transcript and protein sequences of *E.* cf. *sigismundi* and *R.* cf. *coronifer* were compared to sequences from other tardigrades and model organisms. Global comparisons were conducted using BLASTX for the transcripts alignments (left) and BLASTP (right) for the predicted protein sequences. Heatmaps represent the percentage of transcripts (left) or predicted protein (right) with detectable sequence similarity at an e-value threshold of 10e^−5^

In order to investigate and compare the number of shared and unique protein families among a diverse range of eukaryote taxa, we included in the analyses six model organisms with explicit annotations spanning from yeast to human. Firstly, in order to investigate the similarity between the new *E.* cf. *sigismundi* and *R.* cf. *coronifer* transcriptomes and data from other tardigrade and ecdysozoan species, we performed global comparisons of transcripts and predicted protein sequences. As expected, the pairwise global comparison resulted in a high similarity (> 40% for transcripts; > 90% for proteins) of *R.* cf. *coronifer* to that of other eutardigrades (i.e. *H. exemplaris* and *R.* cf. *varieornatus*) (Fig. 1), whereas the similarity of the heterotardigrade *E.* cf. *sigismundi* to the eutardigrade species was lower (< 35% for transcripts; < 75% for proteins). Specifically, for *E.* cf. *sigismundi* the protein similarity with the eutardigrade species was 70.9% to *R.* cf. *coronifer*, 71.7% to *R.* cf. *varieornatus* and 74.9% to *H. exemplaris*. The latter results corroborate the apparent significant divergence of the heterotardigrade and eutardigrade lineages.

**Fig. 2:**
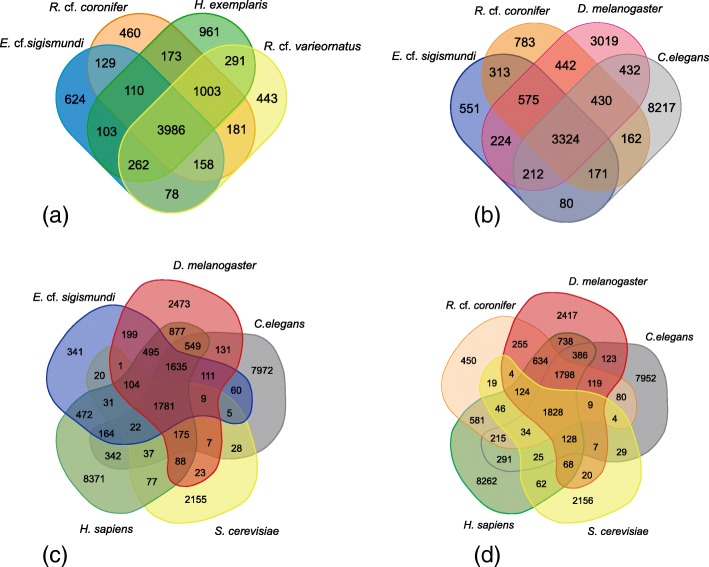
Comparison of shared and species-specific orthologous protein groups as revealed by OrthoMCL analyses. **a** Shared and species-specific orthologous protein groups within tardigrades; **b** Shared and species-specific protein groups between tardigrades and other ecdysozoans as revealed by a comparison between *E.* cf. *sigismundi*, *R.* cf. *coronifer*, *D. melanogaster* and *C. elegans*; **c** Comparison of shared and species-specific orthologous protein groups between *E.* cf. *sigismundi* and four model eukaryote organisms; **d** Comparison of shared and species-specific orthologous protein groups between *R.* cf. *coronifer* and four model eukaryote organisms

To further determine the number of shared and unique protein families among different tardigrades, we constructed orthologous gene clusters using OrthoMCL (default settings) for the four tardigrade species representing four families within the classes, Heterotardigrada and Eutardigrada*.* All-against-all sequence comparisons among tardigrades resulted in the clustering of 79,657 proteins into 8962 orthologous groups (Fig. 2a). When comparing the four tardigrade species (Fig. 2a), almost half of the orthologous groups, 3986 (44%) are common to all species. Heterotardigrada, represented only by *E.* cf. *sigismundi* possess 624 unique orthologous clusters compared to 3512 unique orthologous clusters for the three eutardigrades. Interestingly, the two most closely related species under analysis have, respectively, the smallest (*R.* cf. *varieornatus*: 443) and the largest (*H.* cf. *exemplaris*: 961) number of unique orthologue clusters.

A comparison between our two newly sequenced tardigrades and closely related invertebrates (i.e. the ecdysozoans *D. melanogaster* and *C. elegans*) returned a total of 3324 (17.6%) out of 18,935 orthologous protein groups to be shared between these ecdysozoans (Fig. 2b). Within this comparison, *E.* cf. *sigismundi* has the fewest unique elements, while *C. elegans* appears to have the most. A comparison between *E.* cf. *sigismundi* (Fig. 2c) and *R.* cf. *coronifer* (Fig. 2d) with *D. melanogaster*, *C. elegans* as well as *H. sapiens* and *S. cerevisiae*, revealed that 6% of the orthologous protein groups are shared between these eukaryotes (Fig. 2c,d). Tardigrades, represented by *E.* cf. *sigismundi* (Fig. 2c) and *R.* cf. *coronifer* (Fig. 2d), have the smallest number of unique orthologous clusters, while humans have the largest number, followed by *C. elegans*.

### Comparative analysis of stress related genes

Below we analyze the presence and expression of genes with a previously suggested role in cryptobiotic survival as well as novel candidates with a putative role in adaptations to extreme environmental conditions (see Table 3 and Additional file 1).

**Table 3:** Stress related genes within the ten investigated eukaryotes. Numbers reflect the number of putative genes retrieved within each gene category (See Additional file 1 for details)

Gene class/category	Tardigrada				Arthropoda	Nematoda	Chordata			Ascomycota
	Es	Rc	Rv	He	Dm	Ce	Hs	Xt	Dr	Sc
Tardigrade unique proteins^a^	0	23	32	25	0	0	0	0	0	0
Late Embryogenesis Abundant (*LEA*) ^b^	5	6	9	7	0	2	0	0	0	0
Heat shock proteins^c^	56	65	59	117	79	65	90	77	96	45
RNA/DNA Chaperones_Cold Shock Domain containing^d^	3	1	1	1	1	6	5	5	3	0
DNA repair	(58)	(91)	(74)	(80)	(61)	(57)	(77)	(73)	(74)	(57)
*TP53* ^*e*^	0	1	1	1	1	1	3	3	3	0
Base excision repair^f^	18	31	24	27	15	13	22	21	19	14
Mismatch repair^g^	15	20	13	16	10	12	14	14	14	14
Nucleotide excision repair^h^	18	23	18	21	15	16	18	17	19	15
Non-homologous end-joining^i^	0	5	6	6	8	3	5	5	5	5
Homologous recombination^j^	7	11	12	9	12	12	15	13	14	9
Antioxidative stress	(70)	(79)	(79)	(89)	(82)	(89)	(66)	(53)	(68)	(29)
*Superoxide dismutases* (*SOD_CuZn; SOD_Mn*)	8	14	17	15	6	6	4	2	5	3
*Catalase* (*CAT*)	0	4	4	4	2	3	1	2	1	2
*Peroxiredoxins* (*PRDX*)	5	7	9	12	9	3	6	6	6	3
*Thioredoxins* (*TXN; TXNRD; TXNL; TMX*)	12	13	10	12	14	11	14	12	14	8
*Glutaredoxin* (*GLRX*)	5	4	3	3	4	6	4	4	4	5
*Glutathione-disulfide reductase* (*GSR*)	1	1	1	1	0	1	1	1	1	1
*Glutathione peroxidase* (*GPX*)	2	1	1	1	2	8	8	6	9	3
*Glutathione synthetase* (*GSS*)	1	2	2	3	2	1	1	1	1	1
*Soluble glutathione S-transferases* (*GST soluble*)	35	30	31	34	38	49	22	13	20	2
*Microsomal glutathione S-transferases (microsomal GST)*	0	2	0	2	3	0	4	5	6	0
*Glucose-6-phosphate dehydrogenase (G6PD)*	1	1	1	2	2	1	1	1	1	1
Peroxisomal biogenesis factors^k^	4	3	5	2	17	13	21	21	23	12
Trehalose metabolism	(3)	(9)	(10)	(9)	(5)	(8)	(2)	(2)	(2)	(7)
*Trehalose-phosphate synthase/phosphatase (TPS-TPP)*	0	1	1	0	2	3	0	0	0	4
*Trehalases (PGGHG/ATHL1, TREH)*	3	8	9	9	3	5	2	2	2	3

*Es: Echiniscoides* cf. *sigismundi*, *Rc: Richtersius* cf. *coronifer*, *Rv: Ramazzottius* cf. *varieornatus*, *He: Hypsibius exemplaris*, *Dm: Drosophila melanogaster*, *Ce: Caenorhabditis elegans*, *Hs: Homo sapiens*, *Xt: Xenopus tropicalis*, *Dr: Danio rerio*, *Sc: Saccharomyces cerevisiae*

^a^= *CAHS*; *SAHS*; *MAHS*; *RvLEAM*; *Dsup*

^b^= *LEA*; *DUR-1*

^c^= *HSP90*; *HSP70*; *HSP60*; *HSP40*; *HSP20*; *HSP10*

^d^= *CSP*; *lin28*; *Y-box*

^e^= *Tp53*; *p63*/*p73*

^f^= *UNG*; *XRCC1*; *XRCC3*; *XRCC2*; *PNKP*; *Tdp1*; *APTX*; *POLB*; *POLD*; *POLE*; *FEN1*; *PCNA*; *PARP1–4*

^g^= *MSH2*; *MSH6*; *MSH3*; *MSH4*; *MSH5*; *MLH1*; *PMS2*; *MLH3*; *Exo1*; *RFC*

^h^= *XPC*; *CETN2*; *Rad23*; *DDB*; *GTF2H1*/*TFIIH1*; *GTF2H2*/*TFIIH2*; *GTF2H3*/*TFIIH3*; *GTF2H4*/*TFIIH4*; *CDK7*; *ERCC3*; *ERCC2*; *ERCC1*; *XPA*; *ERCC5*

^i^= *XRCC6*; *XRCC5*; *CLP*/*XRCC4*; *LIG4*; *NHEJ1*

^j^= *MRE11; Rad50; NBS1; Rad51; CtIP; BRCA1; BRCA2; Slx1; SLX4; Mus81; EME1*

^k^= *PEX1; PEX2; PEX3; PEX5; PEX6; PEX7; PEX10; PEX11; PEX12; PEX13; PEX14; PEX16; PEX19; PEX26; PXMP2; PMP34; PXMP4; TYSND1*

### The trehalose pathway

The non-reducing disaccharide, trehalose, has been shown to accumulate to very high concentrations in various anhydrobiotic animals, including nematodes, brine shrimp embryos and sleeping chironomid larvae (see Discussion). Similar high concentrations have not been documented in tardigrades, but trehalose has, nevertheless, been proposed as a possible bioprotectant of membranes and macromolecules during desiccation. Here we investigate and compare the presence of genes with importance for trehalose synthesis and degradation in tardigrades, specifically, trehalose-6-phosphatase synthase (TPS) a key enzyme in the trehalose biosynthetic pathway and the trehalose hydrolyzing enzyme, trehalase (Table 3). No TPS transcripts could be retrieved from *E.* cf. *sigismundi*, however, we found evidence of one homolog in *R.* cf. *coronifer*. Blast search (BLASTP in SWISSPROT and BLASTP and TBLASTN in GenBank) results revealed that the best hits were bacteria with a similarity greater than 40% and e-values <1e-150. Furthermore, alignment with the *R.* cf. *varieornatus* TPS protein sequence revealed high similarity. This result corroborates the findings in *R.* cf. *varieornatus* [17] that suggests a HGT (horizontal gene transfer)-derived TPS. We could not retrieve any TPS homologs from available *Milnesium* cf. *alpigenum* (formerly known as *Milnesium tardigradum*, see [21]) nor *Echiniscus testudo* EST data and *H. exemplaris* is apparently also missing this gene (Additional file 1). All tardigrade species analyzed so far have putative *trehalase* and *acid trehalase* genes. *Trehalase* genes seem expanded in eutardigrades (6–7 putative genes), as compared to most of the other organisms in the analysis including the heterotardigrade, which seemingly only has a single gene (Additional file 1). *H. exemplaris* apparently has an additional *acid trehalase* (3 putative genes) as compared to other tardigrades (2 putative genes).

### Late embryogenesis abundant proteins

Late Embryogenesis Abundant (LEA) proteins prevent protein aggregation during desiccation and have accordingly been identified in a range of desiccation tolerant organisms (see Discussion). Our analyses indicate that LEA sequences are present in all tardigrade species with the eutardigrade lineage showing a higher gene number (6–9 putative genes) compared to the heterotardigrade *Echiniscoides* cf. *sigismundi* (3 putative genes). Only one *LEA* gene could be identified in *C. elegans*, whereas no *LEA* protein encoding genes could be identified in the other organisms*.* Interestingly, in addition to *LEA* genes, *E.* cf. *sigismundi* seems to possess two putative genes of *dur-1* [22]. *Dur-1* exhibits high similarity to *C. elegans* LEA isoforms, as well as LEA isoforms from other nematodes (*Caenorhabditis remanei* and *Caenorhabditis briggsae*), rotifers (*Adineta ricciae* and *Adineta vaga*) and plants. We found no evidence of *dur-1* in the other tardigrade genomes or the EST data of *M.* cf. *alpigenum* and *E. testudo*.

### Tardigrade unique proteins

We continue this analysis of putative cryptobiosis related genes with an investigation of tardigrade unique heat soluble proteins (CAHS, SAHS, MAHS) [15–17] and the mitochondrial RvLEAM [17, 23] (Additional file 1). We found no evidence of expression of any of these genes in the heterotardigrade *Echiniscoides* cf. *sigismundi* and our bioinformatics analyses on several tardigrade species concluded that these genes appear to be present only in the eutardigrade lineage*.* Specifically, we found multiple putative *CAHS* and *SAHS* genes in *R.* cf. *coronifer* (14 = *CAHS* and 7 = *SAHS*), *R.* cf. *varieornatus* (16 = *CAHS* and 13 = *SAHS*) and *H. exemplaris* (12 = *CAHS* and 11 = *SAHS*) and one putative *MAHS* gene and one mitochondrial *LEA* (*RvLEAM*) in each eutardigrade species. We extended our search for these genes to the eutardigrade *M.* cf. *alpigenum*, where we could verify the presence of *CAHS* transcripts in the available EST data [12] as previously reported by [15]. Importantly, we found no evidence of *SAHS*, *MAHS* or *RvLEAM* in this apochelan eutardigrade, indicating that the latter may be specific for the eutardigrade order Parachela. Finally, we did not find any sequence similarity to any of the tardigrade specific proteins in the available EST data of *Echiniscus testudo*, which further corroborates our hypothesis that these genes are in fact eutardigrade and to a large extend parachelan specific.

### Heat shock proteins

A large number of sequences coding for putative heat shock proteins (HSPs) were detected in all of the four investigated tardigrade species. The number of retrieved sequences within different HSP gene families was in accordance within the three eutardigrade species apart, however, from a large gene expansion in the *HSP70* family for *H. exemplaris*, where we retrieved 69 putative genes. Overall, *H. exemplaris* seems to have a highly increased number of HSPs counting up to a total of 117 putative genes, more than any other organism studied herein. *HSP40*s normally represent the family with the highest gene number among all HSPs, thus the very high numbers of *HSP70*s in *H. exemplaris* is exceptional. Curiously, *HSP10* could not be retrieved from *R.* cf. *coronifer*. Interestingly, a comparison on the overall number of putative *HSP* genes reveals that the three strong cryptobiont tardigrade species (i.e. *E.* cf. *sigismundi*, *R.* cf. *coronifer* and *R.* cf. *varieornatus*) have the smallest number of *HSP* genes among the investigated metazoans (Table 3). Among the tardigrades, the marine heterotardigrade *E.* cf. *sigismundi* has the fewest number of putative transcripts and the second lowest number of putative genes, after yeast, among the investigated organisms. Specifically, for *E.* cf. *sigismundi* the number of retrieved transcripts is lower for all categories with the exception of *HSP90*, where *E.* cf. *sigismundi* appears to have more than twice the number of putative genes as compared to the eutardigrades. Overall, *E.* cf. *sigismundi* has the highest number of putative *HSP90* sequences (5) among all organisms analyzed herein, except for humans.

**Fig. 3:**
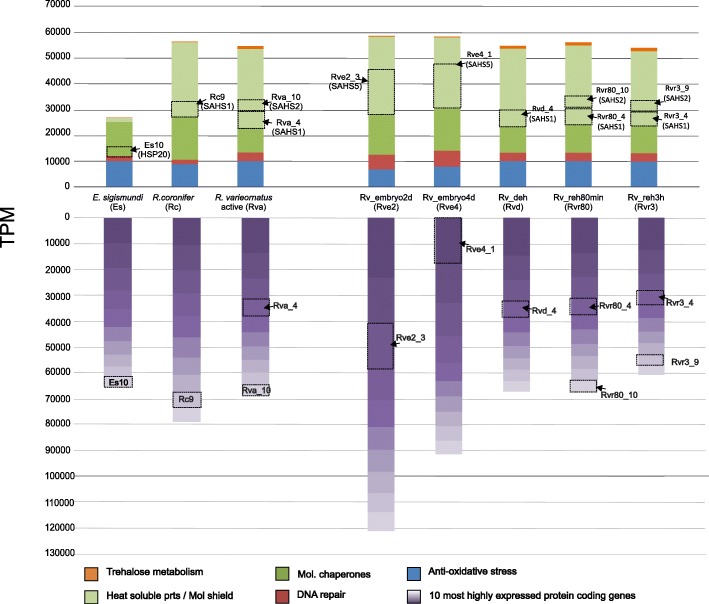
Comparative investigation of gene expression in tardigrades. Comparative data on the 10 most highly expressed protein coding genes within tardigrade transcriptomes and cumulative expression of the stress related gene categories under study (for the complete list of genes refer to Additional file 1). The predicted protein coding genes were obtained from the transcriptomes of three tardigrade species (*Echiniscoides* cf. *sigismundi*, *Richtersius* cf. *coronifer* and *Ramazzottius* cf. *varieornatus*). Values are depicted as TPM. Gradient purple columns: dark = 1st transcript, light = 10th transcript. Rv1,2,3,5,6,7,9 = hypothetical proteins; Rv4 = SAHS1 (Secretory Abundant Heat Soluble 1); Rv10 = SAHS2 (Secretory Abundant Heat Soluble 2); Rv8 = cuticular protein. Rc1,Rc6 = hypothetical proteins; Rc2,5 = uncharacterized proteins; Rc3 = PE-1(Peritrophin-1); Rc4 = rhogef domain containing protein; Rc7 = NOTCH1-like (Neurogenic locus notch-like protein 1); Rc8 = SAHS1 (Secretory Abundant Heat Soluble 1); Rc9 = COI (Cytochrome Oxidase subunit I); Rc10 = periostin. Es1,6,7,8,=hypothetical proteins; Es2,3,9 = uncharacterized proteins; Es4 = CSRP3 (cysteine and glycine rich protein 3 precursor); Es5 = proactivator polypeptide-like; Es10 = HSP20 (Heat Shock Protein 20)

A comparison of *HSP* expression levels among the three tardigrade species with available active stage transcriptomes, reveals that these molecular chaperones have the highest relative expression level in *R.* cf. *coronifer* (Rc = 15,907.5 TPM, Es = 12,359.3 TPM, Rv = 9328.7 TPM). Within the *HSP*s, *HSP20* seems to have the highest expression in all three species [(Es = 4155.5 TPM (4 putative genes); Rc = 6963.8 TPM (6 putative genes); Rv = 2805.1 TPM (8 putative genes)]. For *E.* cf. *sigismundi* the *HSP*s appear to be the most highly expressed gene category of the putative cryptobiosis related genes (Es = 12,359.3 TPM, see Fig. 3 with the highest expressed transcript annotated as a HSP20 (TPM = 4039.2677), followed by one transcript for HSP90 (TPM = 2210.5501) and two HSP70 cognate transcripts (TPM = 1843.26 and TPM = 1838.87).

### Cold shock proteins

**Fig. 4:**
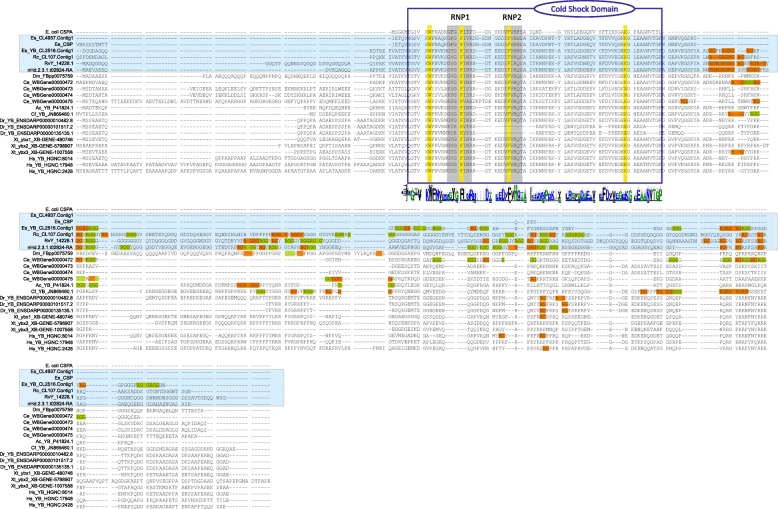
Alignment of amino acid sequences containing a Cold Shock Domain (CSD). Data obtained from representative bacteria and animals including tardigrades. Tardigrade sequences are indicated by bold in the left margin of the figure. RNP1 and RNP2 (shaded in grey) represent consensus RNA binding domains. DNA binding sites are highlighted in yellow. Note the RGG (green) and RG (orange) repeats. In the graphical representation below the CSD, the overall height of the stack indicates the sequence conservation at the specific position, while the height of symbols within the stack indicates the relative frequency of each amino acid at the position

**Fig. 5:**
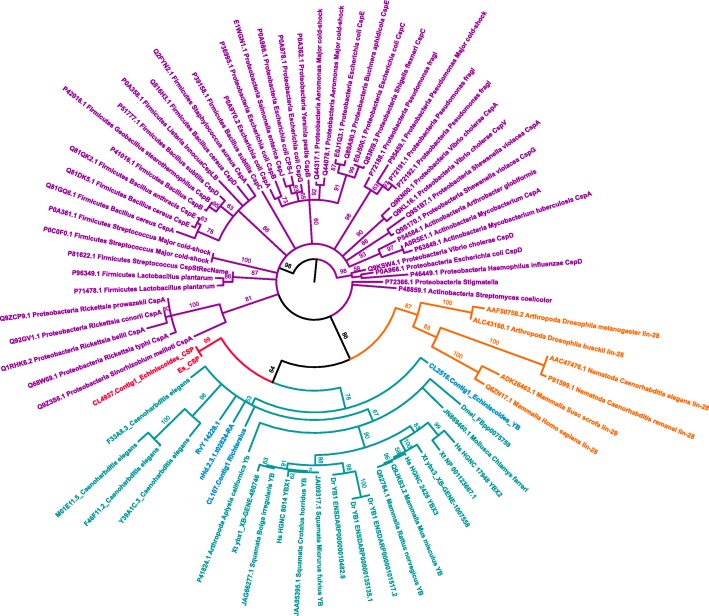
Phylogenetic analysis of Cold Shock Domain proteins. Protein sequences of Cold Shock Domain containing proteins from various species of bacteria and animals aligned using Muscle. The Maximum Likelihood phylogenetic tree was constructed using RAxML. Bootstrap values (1000 trials) are shown on branches. Clades with bootstrap values < 50 have been collapsed into polytomies using TreeGraph2 [92]. All animal taxa are clustered together, and are separated into a clade containing the YB proteins and a clade containing the Lin28 proteins with Zn finger motifs. The Es_CSP form a separate clade as sister-group to the YB cluster of the rest of the animal taxa analyzed

A search for novel candidate proteins related to cryptobiosis and increased tolerance towards environmental extremes revealed the presence of a cold shock domain (CSD), identified as a *Y-box* (*YB*) followed by RGG/RG repeats, in all investigated tardigrade species (Additional file 1). Interestingly, our investigation revealed that tardigrades appear to have more intrinsically disordered RGG/RG repeats in their *YB* sequences compared to other invertebrates (Fig. 4). Another class of eukaryotic CSDs, originally identified in *C. elegans*, i.e. *LIN-28* (with two associated CCHC zinc-finger motifs cooperating CSD target recognition) was found with conserved homologs in *D. melanogaster*, *X. tropicalis*, *D. rerio*, and *H. sapiens*. We could not retrieve *LIN-28* from any of the analyzed tardigrades. In the heterotardigrade, *E.* cf. *sigismundi*, however, besides the *YB* sequence described above, we identified an additional and highly expressed (TPM = 863.974) CSD containing sequence. Interestingly, the predicted protein of this unique sequence lacks the typical invertebrate RGG motifs (Fig. 4). We experimentally validated this aberrant *E.* cf. *sigismundi* sequence by means of PCR (forward primer: 5′-TCA CAA GAG ACA AAC ACC GAA GA-3′, reverse primers: 5′-ATT GAA GAG CAG GAG TGG GG-3′ and 5′-GTT TGG TTG TTG TAG GGC TGA-3′) using a cDNA template generated from the extracted RNA used for the RNAseq analyses **(**GenBank acc. Number: MH500793). In order to evaluate the similarity and evolution of tardigrade CSD containing sequences, Maximum Likelihood phylogenetic analyses were performed using RaxML [24] (CIPRES Science Gateway) and PhyML [25]. Maximum Likelihood robustness was tested by bootstrap analysis with 1000 replicates. The result of the RaxML analysis is presented in Fig. 5. LIN28, *Echiniscoides* CSP and YB proteins cluster in a single large well supported monophyletic clade. All currently identified YB cold shock protein sequences from tardigrades are grouped within the YB clade. The novel *E.* cf. *sigismundi* CSP (Es_CSP) sequences (i.e. two identical sequences retrieved respectively from the transcriptome and generated through PCR) are inferred as sister-group to the YB animal clade. The sister-group position of Es_CSP to the other YB sequences indicates an early separation of the *E.* cf. *sigismundi* Es_CSP sequence from not only the eutardigrades, but also from all other YB animal sequences.

### Antioxidative enzymes

Our analyses reveal that all investigated tardigrade species have a comprehensive number of genes encoding proteins involved in antioxidative stress mechanisms (Table 3, Additional file 1). Here, we investigate the expression and gene copy numbers of enzymes considered important in the defense against reactive oxygen species (ROS).

A comparison of antioxidative enzyme expression levels between active state *E.* cf. *sigismundi*, *R.* cf. *coronifer* and *R.* cf. *varieornatus* show that *soluble glutathione S-transferases* (*GST*s) are the most highly expressed genes in *E.* cf. *sigismundi* (Es = 4988.63TPM from 35 putative genes) and *R.* cf. *varieornatus* (Rv = 4117.3 TPM from 31 putative genes), whereas in *R.* cf. *coronifer CuZn superoxide dismutases* (*CuZn-SODs*) exhibit the highest expression (Rc = 2426.329 TPM; 12 putative genes) followed by *GST*s (Rc = 2216.7808 TPM from 30 putative genes) and *thioredoxin* (Rc = 1393.4664 TPM; 5 putative genes). *CuZn-SOD*s are highly expressed in *R.* cf. *varieornatus* (Rv = 1533.6840 TPM; 16 putative genes), while a somewhat lower expression is seen in *E.* cf. *sigismundi* (Es = 749.3669 TPM; 7 putative genes).

All tardigrade species seem to have a large number of soluble *GST*s known to scavenge superoxide and organic free radicals. Our analyses further reveal that the observed gene expansion in *CuZn-SOD*s previously reported for *Ramazzottius* cf. *varieornatus* [16] seems to be a general tardigrade feature, with eutardigrades possibly having had a second round of duplication (12–16 putative genes) as compared to heterotardigrades (7 putative genes in *E.* cf. *sigismundi*) (Additional file 1). Our data analyses further confirm an apparent expansion within the catalases of parachelan eutardigrades with four putative genes found in all investigated parachelans (*R.* cf. *varieornatus*, *R.* cf. *coronifer* and *H. exemplaris*). Interestingly, as suggested for *R.* cf. *varieornatus* and *H. exemplaris* [16, 17] multiple sequence alignments and Pfam domain searches reveals that *R.* cf. *coronifer* possesses a catalase structure that resembles the bacterial clade II, containing an extra domain annotated as “class I glutamine amidotransferase-like” at the C-terminus. The latter supports the suggestion that parachelan eutardigrades likely obtained their catalase genes through horizontal gene transfer [16, 17]. Our blast searches further confirm a catalase sequence in the EST data from the apochelan eutardigrade *M.* cf. *alpigenum* as reported by [26]. We found no transcripts of catalase in the transcriptome of the marine heterotardigrade *E.* cf. *sigismundi*. On the other hand, reciprocal blast searches against available EST data of the limno-terrestrial heterotardigrade *E. testudo* revealed 8 catalase sequences—using SWISS-PROT we could confirm 5 unique *E. testudo* catalase sequences—indicating a complex picture for the evolution of the catalase gene family within tardigrades.

### Peroxisomal factors

Peroxisomes constitute a key source of reactive oxygen and nitrogen species, but importantly they also counteract oxidative stress containing a variety of ROS metabolizing enzymes. Peroxisomal matrix proteins are transported across the peroxisomal membrane in a folded form aided by a variety of peroxisomal biogenesis factors or peroxins. Our investigation indicates that the cumulative expression level of peroxins is very low in all active state tardigrades (Es = 117 TPM, Rc = 106.4 TPM and Rv = 197.3 TPM). In accordance with previous analysis on *R.* cf. *varieornatus* and *H. exemplaris* [16, 17], we confirm an apparent lack of a large number of peroxins in *E.* cf. *sigismundi* and *R.* cf. *coronifer* (Table 3 and Additional file 1). The loss seems most prominent in *E.* cf. *sigsmundi*, which apparently is missing *PEX2*, *PEX3*, *PEX5*, *PEX7*, *PEX11*, *PEX12*, *PEX13*, *PEX14*, *PEX16*, *PEX26*. On the other hand, *E.* cf. *sigismundi* seems to be the only tardigrade that possesses PEX1 and PEX6 AAA-ATPases indicating a complexity and apparent lineage specificity in the loss of peroxins.

All four tardigrade species appear to be missing peroxisomal membrane proteins (PXMP2, PXMP4, PMP34) and the trypsin domain containing 1 (TYSND1). The main mechanism of direct import of peroxisomal membrane proteins from the cytosol depends on PEX3 (membrane anchor) and PEX19 (cytosolic chaperone). All tardigrades possess *PEX19*, but as noted above *E.* cf. *sigismundi* seems to lack *PEX3*. This is particularly interesting as PEX19 cannot by itself insert membrane proteins.

All tardigrades are missing *PEX7* (PTS2 receptor). In addition, both *E.* cf. *sigismundi* and *H. exemplaris* are apparently missing *PEX5* (PTS1 receptor), considered a core gene in peroxisome protein import pathways. Moreover, all the investigated tardigrade species appear to lack the docking complex consisting of PEX13 and PEX14 required for both PTS1 and PTS2 receptor mediated import and transmembrane channel formation. With respect to Zn RING proteins, we could identify only one (PEX10) for *E.* cf. *sigismundi*, two (PEX10 and PEX12) for *R.* cf. *varieornatus* but none for *R.* cf. *coronifer* and *H. exemplaris*. All tardigrades seem to be missing *PEX11* involved in growth and asymmetric division of pre-existing peroxisomes adding to the overall highly complex picture of tardigrade peroxisomal function.

### DNA damage sensing and repair

DNA damage occurring as a result of endogenous reactive metabolites or various extrinsic factors can potentially pose a serious obstacle to post-cryptobiotic survival. Accordingly, we analyzed the basis for tardigrade DNA repair, based on conserved mechanisms within the eukaryotic DNA damage response (Table 3, Fig. 3, Additional file 1). A total of 57 genes/gene families were included in the analyses. As outlined in Table 3 the highest cumulative number of genes covering these elements was found to be present in eutardigrades with *R.* cf. *coronifer* (91 putative genes) having the highest number of putative genes, followed by *H. exemplaris* (80) and *R.* cf. *varieornatus* (74). When compared to the eutardigrades, *D. melanogaster* and chordates, the heterotardigrade *E.* cf. *sigismundi* seems to express a smaller number of core elements within the different categories of the DNA repair machinery *(*58 putative genes). We state the latter with caution and acknowledge that this finding needs further investigation into genomes and transcriptomes of *E.* cf. *sigismundi* and other heterotardigrades. A low gene count also applies for *C. elegans* (with 57 genes in total), but this does not reflect loss of major gene families as seems to be the case for the heterotardigrade. Cumulative expression levels for the DNA repair elements appear to be low as compared to other transcripts with highest active state expression in *R.* cf. *varieornatus* followed by *E.* cf. *sigismundi* and lastly by *R.* cf. *coronifer* (Fig. 3). Notably, the cumulative expression level in *E.* cf. *sigismundi* derives from approximately 22 less gene copies as compared to the eutardigrades. Below follows a more detailed account of the tardigrade DNA repair machinery (see Additional file 1).

#### The p53 gene family

Members of the *p53* superfamily guard eukaryote genome stability and interact with a variety of DNA-damage-response pathways with at least one *p53* superfamily member (including *p53* and *p63/p73-*like genes) identified in all animal species studied so far. We could not identify any *p63/p73*-like homologues in the tardigrade species under study; however, we retrieved one homologue of *p53* from all investigated eutardigrade species (Table 3, Additional file 1). When compared to human *TP53,* eutardigrade *p53* homologues show a sequence similarity of respectively 28% (*R.* cf. *varieornatus*), 23% (*R.* cf. *coronifer*) and 20% (*H. exemplaris*). We found no homologues of the *p53* gene in the transcriptome of *E.* cf. *sigismundi*. To our knowledge, this is the first record of an ecdysozoan apparently lacking all three members of the *p53* family. We state the latter with caution and acknowledge that this finding needs further investigation.

#### DNA repair mechanisms

Within the *Base Excision Repair* pathway, we find that eutardigrades seem to have a larger number of putative genes as compared to the other organisms under investigation. Especially, *R.* cf. *coronifer* seems to have more copies within a number of gene families (*POLE*, *FEN1*, *PARP1–4*), when compared to all other investigated organisms (Table 3, Additional file 1). Interestingly, all tardigrades have at least one more copy of *POLE* (*polymerase epsilon*) as compared to other organisms, with *R.* cf. *coronifer* having eight putative genes. On the other hand, the heterotardigrade *E.* cf. *sigismundi* lacks *POLB* (this gene is also missing in *D. melanogaster* and *C. elegans*) and *aprataxin* (*APTX*), which is missing in *C. elegans* as well. In contrast to *E.* cf. *sigismundi*, the eutardigrades have an additional copy of *APTX* as compared to the other organisms in our analyses. An investigation into genes involved in *Mismatch Repair*, a mechanism responsible for post-replicative repair of errors that escaped the DNA polymerase, repair of mismatches that arise as a consequence of genetic recombination, but also DNA damage, indicates that tardigrades, basically, have the same inventory as other organisms (Table 3, Additional file 1). They, however, all lack *MSH3* (and thus MSH3 heterodimers) as also holds for the other ecdysozoans in our analyses, i.e. *D. melanogaster* and *C. elegans*. Additionally, *E.* cf. *sigismundi* seems to lack the *EXO1* gene. All investigated tardigrades seem to have the central components of *Nucleotide Excision Repair,* a highly versatile repair pathway that recognizes and removes a wide variety of bulky, helix-distorting lesions from DNA, e.g. products of UV-irradiation. Some of the gene families within this pathway appear to be expanded (e.g. *CDK7*, *ERCC1*, *ERCC5* and general transcription factors*—*see Table 3 and Additional file 1). We identified multiple transcripts encoding the general transcription factor IIH subunit 1, 3 and 4 in *R.* cf. *coronifer*, however, these sequences were short (< 300 bp) and thus omitted from the analyses, hence we refer to the particular gene copy numbers with caution.

*DNA double-strand breaks* (DSBs) are the most deleterious form of DNA damage that, if left un-repaired or incorrectly repaired, may result in massive loss of genetic information, genomic rearrangements and cell death. Here we investigate the basis for DSB repair by classical *Non-Homologous End Joining* (c-NHEJ) and *Homologous Recombination* (HR) in tardigrades. c-NHEJ is considered highly conserved throughout eukaryotes both in terms of function and gene copy numbers. The most prominent divergence of the heterotardigrade *E.* cf. *sigismundi* from the other investigated organisms, including eutardigrades, was found within c-NHEJ, as this tardigrade appears to completely lack the entire c-NHEJ mechanism (Table 3, Additional file 1). Specifically, we were unable to identify any of the essential c-NHEJ components (see Additional file 1) within the *E.* cf. *sigismundi* transcriptome. In addition, we found no evidence of c-NHEJ promoting *53BP1* homologues in the *E.* cf. *sigismundi* transcriptome, whereas we identified seven transcripts of this p53 binding protein in *R.* cf. *coronifer* (data not shown). The latter provides additional evidence of a lack of the entire c-NHEJ mechanism in the heterotardigrade, whereas eutardigrades seem to have the essential components of this pathway. Interestingly, the end resection and HR promoting *BRCA1* gene only seems to be present in *E.* cf. *sigismundi*, while we could not retrieve *BRCA1* from any of the eutardigrade species (*R.* cf. *coronifer*, *R.* cf. *varieornatus* and *H. exemplaris*). The latter findings indicate highly divergent use of pathways associated DSB repair among tardigrade lineages.

The HR mediator of recombinase RAD51 recruitment to DSBs, BRCA2, appears to be missing from all tardigrades under analyses. Whereas many HR components are present in tardigrades, all investigated species appear to lack central elements of the pathway identified in human cells (and yeast) where initiation of DNA end resection is mediated by a MRN (*Mre11*-*Rad50*-*NBS1*) complex facilitated by *CtIP*. We did not find *NBS1* sequence homologues in any of the tardigrade species under investigation and an extension of the analyses to include available EST data from *M.* cf. *alpigenum* and *E. testudo* confirmed a lack of *NBS1* in tardigrades, as holds for the nematode *C. elegans*. Additionally, no *CtIP* sequences were retrieved from tardigrades indicating an aberrant switch between c-NHEJ (when present) and HR mechanisms with a possible use of HR in non-dividing cells as would be expected for adult tardigrades that have very low levels of mitotic activity. An additional peculiarity found in the heterotardigrade *E.* cf. *sigismundi* is the apparent absence of *SLX1* and *SLX4* genes with possible implication for Holliday Junction resolution. As noted above *E.* cf. *sigismundi* further seems to lack *EXO1*, but also *MLH3* is lacking and thereby a possible *MutLγ* (*Mlh1*-*Mlh3*) and *Exo1* containing junction resolution pathway. It would thus appear that this heterotardigrade utilizes a different way than the eutardigrades for dissolving the Holliday Junctions after homologous recombination.

## Discussion

### Global comparison of tardigrade gene content

A comparison of the overall gene content of our two new transcriptomes against genomes of other tardigrades, as well as six eukaryote model species, reveals that tardigrades possess a genetic toolkit corresponding to key metazoan features and functions (Fig. 2). Our analyses show relatively few unique orthologous elements within the tardigrades, which could indicate major gene losses within the phylum as argued by e.g. [27]. Within the tardigrades, the unique orthologous clusters present in different species stresses the divergence present within the phylum. *H. exemplaris—*a weak cryptobiont [28] and the least stress tolerant among the four investigated tardigrade species*—*has the highest number of unique orthologous. It thus seems unlikely that cryptobiosis is related to a high number of unique elements. On the contrary, if the number of orthologous groups has a bearing on functional traits, this would according to our current analyses indicate that “less is more”, when it comes to being a strong cryptobiont. Our comparative analyses of stress related gene families returned evidence of a lack of expression of key elements in the heterotardigrade *E.* cf. *sigismundi* and several gene expansions that are more frequent within the eutardigrades. The latter observation could potentially*—*to some extent*—*reflect a difference in habitat, as *E.* cf. *sigismundi* is the only marine species included in this comparison. Below we discuss in more detail the obtained results and their possible significance for cryptobiotic survival.

### Trehalose metabolism

Disaccharides like trehalose and sucrose, as well as glycerol and other sugars have been suggested to have a prominent role in cryptobiosis with empirical evidence gained from studies on anhydrobiosis. The sugars protect membranes and macromolecules from desiccation damage by replacing water and by forming amorphous glasses (vitrification) that trap biomolecules and maintain their structures in time and space preserving them from desiccation damage [29]. Trehalose has been found to accumulate to extremely high concentrations (up to 10–20% wt/dry wt.) in several anhydrobiotic animals, including nematodes [30], embryos of the crustacean *Artemia salina* [31], and the insect larva *Polypedilum vanderplanki* [32]. In contrast, no trehalose was detected in the anhydrobiotic bdelloid rotifers [33]. In the case of tardigrades the highest trehalose concentrations measured in anhydrobiotic animals range from 2.3% d.w. in *Richtersius* cf. *coronifer* and *Macrobiotus krynauwi* to 2.9% d.w. in *Diaforobiotus islandicus* [34, 35]. Trehalose accumulation during dehydration was reported in several parachelan species [36], whereas no change in trehalose level was detected in heterotardigrades and Jönsson and Persson [35] found no changes in levels of trehalose between active and anhydrobiotic individuals of *Milnesium* cf. *alpigenum.* Our current data on tardigrade gene content confirm previous suggestions that trehalose accumulation alone cannot explain cryptobiotic survival [1, 35]. Specifically, our data on genes involved in trehalose metabolism support the observation that not all tardigrades possess the TPS gene [17]—and that those parachelans that do have it, appear to have acquired it through horizontal gene transfer. Obviously, future analyses of more tardigrade genome datasets are required to substantiate this observation.

### Molecular shields and chaperones

It has been proposed that the intrinsically disordered LEA proteins act as molecular shields preventing protein aggregation during desiccation and that LEAs further may function as membrane protectants, ion sinks, hydration buffers and antioxidants [37–40]. It has, moreover, been argued that the stabilizing capacity of LEAs during desiccation is enhanced by the presence of sugars, such as trehalose [12, 41, 42]. Recent data on *R.* cf. *varieornatus* revealed that LEA genes were constitutively expressed between active, dehydrated and rehydrated tardigrades with a higher expression in embryos compared to adults [16]. Our analyses show that LEA sequences are present in all four tardigrade species with the eutardigrade lineage showing the highest gene numbers, supporting the assumption that LEA proteins may exhibit diverse protective abilities within the tardigrades. Hashimoto and coworkers [16] further reported that tardigrade unique proteins CAHS, SAHS and MAHS are constitutively expressed before, during and after dehydration in *R.* cf. *varieornatus*. Our analyses of expression data from the active stage of *R.* cf. *coronifer* reveal that a *SAHS* gene appear to be the most highly expressed gene among all the genes previously implicated in cryptobiosis, followed by *CAHS*. The high expression levels found in the current study for the active stage of *R.* cf. *coronifer* would indeed suggest that these proteins are important for the physiology of eutardigrades, but our present investigation does not support a direct dependence on these proteins for cryptobiotic survival in general as suggested by [43]. Specifically, *E.* cf. *sigismundi*, a strong cryptobiont with extreme resilience against a variety of stresses (dehydration, freezing, environmental toxicants, osmotic stress) [1, 18, 44, 45] seems to be lacking all the previously identified tardigrade unique proteins (i.e. CAH, SAHS, MAHS, RvLEAM).

A large number of putative *HSP* genes were detected in all investigated tardigrade species. HSPs act as molecular chaperones with important roles in preventing protein aggregation, supporting refolding of denatured proteins and degradation of aberrant proteins [46, 47]. *HSP* expression can be induced by many environmental stressors apart from heat, also cold, food depletion, osmotic stress and toxicants, however, experimental studies on HSP expression in tardigrades have returned contradicting results regarding the putative role of these proteins in relation to cryptobiosis (summarized in [1]). As also noted by [17], the very high numbers of *HSP70* genes in the eutardigrade *H. exemplaris* is exceptional. We hypothesize that the apparent gene expansion within HSP70 in *H. exemplaris* is related to the limited cryptobiotic capability of this tardigrade, thus representing an alternative survival strategy as compared to immediate metabolic shut-down characteristic of environmental induced cryptobiosis. This tardigrade may be utilizing the cooperation of the extensive number of HSP70s together with other chaperone systems to broaden their activity spectrum generating a complex network of protein folding machines.

### Cold shock domain

Cold shock domain (CSD)-containing proteins have been found in all three domains of life. These proteins function in a variety of processes that are related, for the most part, to stress linked post-transcriptional gene regulation, e.g. involved in adaptation to low temperatures [48, 49]. Here, we report, for the first time, the presence of a core animal cold shock domain identified as a Y-box (YB), followed by RGG/RG repeats, in all investigated tardigrade species (Fig. 4). Importantly, our investigation revealed that tardigrades appear to have more intrinsically disordered and likely post-translationally modified (arginine methylation) RGG/RG repeats in their YB sequences as compared to other invertebrates. The latter finding indicates that epigenetic regulation may be an important, but at present an unexplored factor in cryptobiotic survival. Interestingly, in the heterotardigrade, *E.* cf. *sigismundi*, in addition to the more typical metazoan CSDs, we found an additional highly expressed bacteria-like CSD, lacking RGG/RG repeats. The fact that the Es_CSP transcript is very long, but most of it is non-coding (Additional file 2), could suggest that it originally was an YB-like protein, which acquired a premature stop codon, thereby losing the last part of the protein. *E.* cf. *sigismundi* is a marine tidal tardigrade with an extreme tolerance towards a range of environmental stresses, including freezing. We hypothesize that the Es_CSP (which is only 86 amino acids long) has a function similar to that of bacterial CSPs. Specifically, the CSPs in *E. coli* are induced from cold shock and are required for growth in low temperature [50]. In *E. coli* the CspA protein increases translation of its own mRNA. The non-specific binding of CSPs to RNA prevents the formation of secondary structure, thus keeping the mRNA in a linear form essential for translation [50].

### Oxidative stress

A common feature of environmental stress, such as heat, cold, dehydration, osmotic shock and UV-radiation, is the generation of free radicals and change in cellular redox potential [51]. Cell damage is typically caused by the accelerated production of ROS, such as superoxide, hydrogen peroxide, and the highly reactive hydroxyl radical, leading to a deregulation of redox sensitive pathways as well as oxidative modification of essential biomolecules (e.g. DNA, proteins, lipids) [52–54]. Tardigrades have been proposed and also shown to upregulate antioxidant defense mechanisms during cryptobiosis [26, 55–57] and a well-developed antioxidant defense system has been suggested as a possible explanation for the highly increased radiation tolerance seen among tardigrades [16, 58]. Our analyses support the above hypotheses revealing that all investigated tardigrade species have a comprehensive number of genes involved in antioxidant defense. Most conspicuously, all investigated tardigrades exhibit gene expansions within the Cu-Zn superoxide dismutases likely located within mitochondria, cytosol, but also peroxisomes.

Catalases can be located in all major cellular compartments, preventing H_2_O_2_ accumulation by its removal in a catalatic or peroxidatic manner [59]. Catalase sequences are conserved within metazoans, yeast and plants*—*additionally, some obligate anaerobes are known to contain typical catalases (e.g. species of the genera *Clostridium* and *Bacteroides*, *Methanobrevibacter arboriphilus*) [60]. Intriguingly, despite their apparent importance, catalases are not essential for life [59] and there are examples of eukaryote organisms lacking these enzymes (e.g. *Euglena* spp., *Neurospora crassa*) [61]. It has, furthermore, been documented that catalase null mice develop normally and do not display any gross physical or behavioral abnormalities [62]. Our data suggest that the catalase gene family indeed seems expanded within the parachelan eutardigrades (4 putative genes), and that a similar high number of catalase sequences may be present in the heterotardigrade *Echiniscus testudo*. On the other hand, the apochelan eutardigrade, *Milnesium* cf. *alpigenum*, apparently only has a single copy of this gene and the marine heterotardigrade *E.* cf. *sigismundi* seemingly lacks catalases entirely. Our analyses support the suggestion that parachelan eutardigrades possess a catalase structure resembling bacterial clade II catalases, revealing a complex evolutionary scenario for tardigrade catalases and at the same time seemingly confirming that functional stress related HGT genes are present in tardigrades. Interestingly, *E.* cf. *sigismundi* presumably utilizes alternative genes in the battle against the deleterious effects of free radicals. Importantly, all investigated tardigrade species have a large number of soluble glutathione S-transferase (GSTs)—a large group of multifunctional enzymes localized throughout the cell that scavenge superoxide and organic free radicals and act as substrate in enzymatic reduction reactions. GSTs are induced upon a variety of stressors; they are characterized by wide substrate specificity, high activity and inducibility in response to various toxicants. The ubiquitous occurrence of GSTs among tardigrades indicates that enzymes of this group play a fundamental role in tardigrade antioxidant defense.

It has been proposed that animals, which suppress metabolism in response to stress have the advantage of a slow metabolic recovery decreasing the production of ROS and thereby limiting cellular and genomic damage [63]. A complete metabolic shutdown, preventing ROS generation from internal sources, coupled with apparent species-specific adaptations within antioxidant defense systems could explain how tardigrades seemingly diminish the deleterious effects of ROS damage and thereby ensure post-cryptobiotic survival [54, 58].

### Peroxisomes

The peroxisome is a ubiquitous eukaryotic cell organelle that participates in a wide variety of metabolic, signaling and developmental processes co-operating extensively with other organelles, especially mitochondria [64, 65]. Peroxisomes constitute a key source of cellular ROS/RNS, but importantly they also possess well-developed protective mechanisms that counteract oxidative stress and maintain redox balance. Specifically, these organelles may contain a wide variety of ROS metabolizing enzymes. Peroxisomal proteins are encoded by nuclear genes and ultimately transported across the peroxisomal membrane aided by a variety of peroxins. Peroxisomes can be formed either by growth and asymmetric division of pre-existing organelles*—*a process that is regulated by the *PEX11* gene family*—*or alternatively they can be formed de novo from the ER. We could not retrieve any putative transcripts of *PEX11* in any of the investigated tardigrade datasets, thus tardigrades could be forming peroxisomes de novo from the ER. Our analyses show a major loss of peroxins within all investigated tardigrades including loss of components involved in the predominant PEX5-mediated import pathway.

Peroxin silencing experiments in *C. elegans* have shown that defective peroxisome biogenesis affects early development, however, no deleterious effects have been documented for adults; on the contrary, silencing of *PEX5*, *PEX11*, *PEX13* and *PXMP4* in adult worms triggered an extension of life span suggestively related to a verified reduction in ROS [59, 66]. Peroxisome metabolic functions may vary between organisms and tissues, depending on developmental stages and changed environmental conditions [67]. Overall, it remains unclear if and how tardigrades utilize peroxisomes. Specifically, tardigrades appear to have extensive losses within several of the peroxisome biogenesis categories. It could be speculated that these losses may enable tardigrades to manipulate the use of peroxisomes “on demand”, e.g. during recovery from stress induced states, such as cryptobiosis.

### Tardigrade DNA damage sensing and repair

A failure to repair damage to DNA obtained while in an ametabolic cryptobiotic state may alter or even eliminate fundamental processes, such as DNA replication and, for tardigrades with little adult mitotic activity, transcription. One of the main hypotheses proposed in order to explain the impressive cryptobiotic capabilities of tardigrades is an extreme efficiency in DNA repair mechanisms [12, 68–70]. Whereas DNA damage following cryptobiosis has been documented in tardigrades [68], detailed knowledge of the tardigrade DNA repair apparatus has been lacking. As discussed in more detail below, our investigation points to a possible vital role for transcription-coupled repair. Our results, however, also reveal a clear divergence between the two major tardigrade lineages as evidenced by selective gene expansions and apparent highly conspicuous losses.

Our analyses show that tardigrades seem to encode most components of the mismatch repair system and all of the components of the central nucleotide excision repair (NER) pathway discovered in vertebrate cells, with some of the gene families appearing to be expanded. Notably, transcription-coupled*-*NER, plays an important role in removal of lesions from template DNA strands of actively transcribed genes [71]. The latter is in accordance with the observation that adult tardigrades seem to have low rates of mitotic cell divisions limited to specific cells and tissues [72, 73], calling for a method of DNA repair that does not rely on replication. Our analyses indicated a conspicuous lack of members of the *p53* family in the heterotardigrade *E.* cf. *sigismundi*. This species also seems to lack DNA polymerase POLB involved in Base Excision Repair (BER). We hypothesize that *E.* cf. *sigismundi* has retained only the necessary amount of these error-prone translesion enzymes, which potentially may jeopardize BER fidelity due to high misincorporation rates. POLB is also missing in *D. melanogaster* and *C. elegans*, indicating that loss of this polymerase has occurred several times during ecdysozoan evolution. Most conspicuously, *E.* cf. *sigismundi* appears highly divergent in regard to mechanisms involved in repair of double-strand breaks (DSBs) apparently lacking the entire c-NHEJ system. While this manuscript was under review, a study from Deng and coworkers, 2018 [74] was published, reporting lack of c-NHEJ genes in the genome of the tunicate *Oikopleura dioica* as well as a number of other tunicates [74]. The authors, however, experimentally validated the utilization of other conserved genes (*Parp1*, *Mre11*, *XRCC1*, *RAD50*, and *Lig1*) in a possible alternative NHEJ-like mechanism. The latter genes are present in the *E.* cf. *sigismundi* transcriptome, and this organism could thus be utilizing similar mechanisms in order to compensate for the potential lack of the c-NHEJ mechanism. Interestingly, emerging evidence suggest that in non-dividing cells, HR appears to be coupled to transcription, using RNA as template [75]. We propose that transcription-coupled HR could be an important method for high fidelity post-cryptobiotic DNA repair in tardigrades.

## Conclusions

The cryptobiotic capabilities of tardigrades differ between species and it has been proposed that the variety of molecular functions is richer in species that exhibit increased tolerance towards environmental stress [12]. Our analyses seem to contradict this hypothesis and could suggest that for tardigrades “less is more”, as strong cryptobionts seem to express less genes in several of the proposed cryptobiosis related categories. Specifically, we retrieved smaller numbers of putative genes for the strong cryptobiont *E.* cf. *sigismundi* in almost all the investigated gene families and this heterotardigrade appears to be lacking the previously highlighted tardigrade specific heat soluble proteins (CAHS, SAHS, MAHS, and RvLEAM; Table 3, Additional file 1). Curiously, *E.* cf. *sigismundi* is apparently also lacking a number of important transcripts within DNA repair mechanisms, including p53, aprataxin, POLB and all essential c-NHEJ components. We hypothesize that *E.* cf. *sigismundi* utilizes alternative paths of repairing deleterious double strand breaks and that tardigrades have a preference for DNA repair mechanisms with high fidelity. We propose that the low level of cell divisions in adult tardigrades combined with an ability to repair DNA during transcription might be of vital importance for post-cryptobiotic survival.

All analyzed tardigrade species appear to have been subjected to extensive gene loss within the peroxisome biogenesis, division, recycling and quality control categories. On the other hand, within the antioxidative enzymes, all species show an expansion of SODs underlining the importance of antioxidant defense for these animals. Furthermore, our transcriptome of *R.* cf. *coronifer* corroborates the expansion of HGT-derived catalases and a HGT-derived TPS within parachelan eutardigrades*.*

We retrieved a highly expressed transcript from a novel Cold Shock Protein in *E.* cf. *sigismundi*. The aberrant sequence could be derived from an ancestral YB-like cold shock gene that also gave rise to the animal YB clade. On the basis of similarity to bacterial CSP sequences (i.e. residue composition, short sequence length and lack of the typical metazoan RGG/RG repeats) we hypothesize that this highly expressed CSP could have a function similar to that found in bacteria. Specifically, the CSP in *E. coli* is induced from cold shock and subsequently increases translation of its own mRNA and prevents the formation of secondary structure, thereby keeping the RNA in a linear form required for translation.

Our results thus indicate that different tardigrade taxa and lineages exhibit unique adaptations towards stress involving possible unknown functional homologues. An interesting characteristic that deems further investigation is the level of abundance of low-complexity and intrinsic disordered regions, which potentially could hold important clues towards the understanding of cryptobiosis and extreme stress tolerance. Our transcriptomes provide preliminary evidence of the possible importance of such sequences; specifically, in *E.* cf. *sigismundi* an unannotated/novel transcript with the second highest TPM value (Fig. 3) exhibits high disorder as holds true for a number of other highly expressed transcripts (data not shown). The “tardigrade unique proteins” were indeed proposed to be disordered [15, 43]. We further propose that the unique adaptations found among tardigrades and other cryptobionts involve or may indeed to a large extent also rely on epigenetic regulation. It is highly likely that post-transcriptional and post-translational mechanisms are involved in the stress response affecting proteome and metabolome complexity via fine tuning of both the amount and activity of pre-existing transcripts and proteins.

## Methods

### Tardigrade sampling and RNA extraction

Marine heterotardigrades, *Echiniscoides* cf. *sigismundi,* were collected from barnacles in the intertidal zone at Lynæs, Zealand, Denmark (see [57]). Specimens of a parthenogenetic population of the eutardigrade, *Richtersius* cf. *coronifer*, were sampled from moss growing on limestone in Øland, Sweden (see [76]). Total RNA was extracted from active stage pools of ∼550 *E.* cf. *sigismundi* and ∼200 *R.* cf. *coronifer*, respectively, using a RNeasy Plus Universal Mini Kit (Qiagen,Hilden, Germany). RNA quantitation and quality analyses were performed using a NanoDrop ND-1000 (Thermo Scientific, Waltham, Massachusetts, USA) and a Bioanalyzer 2100 (Agilent Technologies, Santa Clara).

Cryptic species complexes are common within Tardigrada and the genus *Echiniscoides* has an exceptionally large genetic variation [19]. We therefore analyzed both transcriptomes with respect to the often used barcoding sequence of cytochrome c oxidase subunit I (*COXI*) using BLASTN searches in the NCBI nucleotide non-redundant database. The transcriptome data revealed tardigrade *COXI* contigs (CL4672.Contig1_Echiniscoides and CL1502.Contig2_Richtersius), which support that the 550 *Echiniscoides* and 200 *Richtersius* specimens each constitute single species. BLASTN searches in GenBank returned a 99% identity to the isolate *Echiniscoides* cf. *sigismundi* (voucher ZMUC:TAR Esi1; GenBank Accession number: HM193403.1; [77]. Similarly, the *Richtersius* cf. *coronifer* BLASTN search in GenBank returned a 99% identity to the isolate *Richtersius* cf. *coronifer* (GenBank Accession number: EU244606.1).

### Transcriptome sequencing, de novo assembly and annotation

Paired-end (2 × 100) strand specific transcriptome sequencing (TruSeq RNA-Seq) using Illumina Hiseq 2000 technology and basic bioinformatic analyses were conducted by BGI (BGI Tech Solutions Co. Shenzhen, Guangdong, China). In total, ca. 122 million pairs of paired-end reads were generated. One library for the pooled individuals was generated for each species. After vector-clipping, trimming and quality checking of the raw sequences, transcriptome de novo assembly was conducted using the Trinity software package [78] setting the parameter ‘--min_kmer_cov’ to 4. The Trinity software uses the term “Unigene” for uniquely assembled transcripts. These Unigenes do not necessarily correspond to a specific gene as several transcripts may have been assembled for the same gene. Transcript abundance within each transcriptome was measured using FPKM values (Fragments per kb of transcript per million mapped fragments [79, 80]. We have subsequently converted FPKM values to TPM (Transcripts per million) using TPM = [FPKM/FPKM_sum_]·10^6^ [81]. The BGI de novo assembly generated 55,499 contigs for *E.* cf. *sigismundi* and 84,106 contigs for *R.* cf. *coronifer* that clustered into 31,601 and 55,053 non-redundant Unigenes respectively. Summary statistics for both transcriptomes are presented in Table 1.

Annotation analyses of each library was conducted by matching to public protein and nucleotide databases. Specifically, Unigenes were blasted (BLASTX, *e*-value <10e-05) against the NR non-redundant NCBI protein database, Swiss-Prot, KEGG, COG and against the NT non-redundant nucleotide NCBI database (BLASTN, *e*-value <10e-05). Blast2GO [82] was used in order to attribute Gene Ontology terms to the Unigenes. The classification into GO ontologies: molecular function, cellular component and biological process, was conducted with WEGO (The Gene Ontology Consortium 2015). In total, 14,159 (44.8%) genes in *E.* cf. *sigismundi* and 20,326 (36.9%) in *R.* cf. *coronifer*, were annotated. The remaining sequences did not reveal any significant hits in any of the aforementioned databases and could potentially be considered novel transcripts, untranslated regions, non-coding RNA or short sequences not containing a protein domain. The annotation statistics for both transcriptomes are summarized in Table 2.

### Transcriptome quality and completeness

We used CEGMA (Core Eukaryotic Genes Mapping Approach) [83] and BUSCO (Benchmarking Universal Single-Copy Orthologs, [84]) libraries for Eukaryota and Metazoa orthologous genes in order to evaluate the completeness of the assemblies. For *E.* cf. *sigismundi*, the CEGMA analysis revealed that of 248 ultra-conserved eukaryotic genes, 233 were found as complete gene sequences and 7 were found as partial gene sequences. As reported in [57], this gives a recovery percentage of 96.8%, which is comparable to other published transcriptomes, and verifies that the obtained transcriptome database is of high quality. From the additional BUSCO analysis using orthologues of Eukaryota, we obtained 280 (92%) complete (242 single-copy and 38 duplicated) and 10 (3.3%) fragmented hits out of 303 groups, which gives a cumulative recovery of 95.3%. When using the Metazoa library the recovery percentage was 86% [806 complete (691 single-copy and 115 duplicated] and 34 fragmented hits out of 978 BUSCOs). For *R.* cf. *coronifer* the same analyses returned a recovery percentage of 98% via CEGMA (233 complete and 10 fragmented hits), 97.7% via BUSCO Eukaryota [285 complete (212 single-copy and 73 duplicated) and 11 fragmented hits] and 91.4% via BUSCO Metazoa [853 complete (624 single-copy and 229 duplicated) and 41 fragmented hits]. In both analyses, the number of complete and duplicate genes that were recovered provides an important validation of the depth and completeness of the assembly.

### Comparative tardigrade transcriptomics and putative cryptobiosis-related gene analyses

Available sequence data from *Hypsibius exemplaris* [14], *Ramazzottius* cf. *varieornatus* [16], *Drosophila melanogaster* [85] (Flybase: http://flybase.org/), *Caenorhabditis elegans* (Wormbase http://www.wormbase.org/), *Xenopus tropicalis* [86, 87] (http://www.xenbase.org/), *Danio rerio* [88] (https://zfin.org/), *Homo sapiens* (*HUGO Gene Nomenclature Committee at the European Bioinformatics Institute,* http://www.genenames.org/*) and Saccharomyces cerevisiae* [89] *(*http://www.yeastgenome.org/) were retrieved and used for a global comparison of our two new transcriptomes in terms of similarity, orthologue genes, and functional categories. We performed global comparisons using BLASTX for the transcripts alignments and BLASTP for the predicted protein sequences. The percentages of similarity between the various species were visualized as heatmaps using *heatmap* in Rstudio. The orthologous gene groups were further investigated using the OrthoMCL algorithm [90, 91] with default parameters (10e-05, protein identity 50%, and MCL inflation of 1.5).

Tardigrade transcriptomes were investigated for sequences with a putative role in cryptobiosis. In total, 107 gene families were investigated. Individual sequence sets were created for each gene using the relevant amino acid sequences from *R.* cf. *varieornatus*, *D. melanogaster* and *C. elegans* and used as a guide in order to retrieve the relevant transcripts from the two new transcriptomes. TBLASTN and BLASTP (CLC main workbench 6, CLCbio, Århus, Denmark) was used in order to investigate the transcriptomes of *E.* cf. *sigismundi* and *R.* cf. *coronifer*. Using the same methodology we also screened the available data from *H. exemplaris* (files used: nHd.2.3.1.aug.transcripts.fasta and nHd.2.3.1.aug.proteins.fasta; [14]) and *R.* cf. *varieornatus* (files used: RvY_cds_scaf199.fa and RvY_proteins_scaf199.fa; [16]. The specific sequences identified in the two new transcriptomes were verified through reciprocal BLAST searches against NCBI/SWISSPROT (cutoff *e*-value <10e-5). The hits were subsequently mapped back on the respective gene sequences in order to check whether they constituted parts of the same gene. All unique transcript hits might not necessarily represent individual genes, since transcriptomic assemblies can contain sequences belonging to both overlapping and to non-overlapping fragments of the same gene, thus all unique blast hits cannot necessarily be perceived as individual genes. We further applied a filter using R scripts to i) retain Unigenes that had an FPKM value > 1, ii) discard Unigenes that were shorter than 300 bp long and iii) keep the longest contig per locus, thereby discarding e.g. splice variants and very short sequences, but also transcripts with a very low expression level that potentially could reflect contaminations. After the compilation of the list containing the identified genes with a putative role in cryptobiosis (See Additional files 2 and 3), we expanded the comparative analysis to include *X. tropicalis*, *D. rerio*, *H. sapiens and S. cerevisiae* in order to have an overview of the gene content within the relevant gene categories throughout a wide spectrum of evolutionary lineages. The aforementioned databases on each species were searched for the genes of interest and the orthologues of the six model species were checked using the catalog of orthology predictions for model organisms in both HGNC and FLYBASE.

Finally, we compared expression patterns of the selected genes between the two new transcriptomes and transcriptome data from different stages (two embryonic stages, active, desiccated and two rehydrated stages) of *R.* cf. *varieornatus* [16]. In order for the expression data to be comparable between the three datasets, we converted the FPKM values for each transcript/gene to TPM [81].

## Additional files

Additional file 1: Comparative analysis of stress related genes among 10 species. Es = *Echiniscoides* cf. *sigismundi*, Rc = *Richtersius* cf. *coronifer*, Rv = *Ramazzottius* cf. *varieornatus*, He = *Hypsibius exemplaris*, Ce = *Caenorhabditis elegans*, Dm = *Drosophila melanogaster*, Xt = *Xenopus tropicalis*, Dr. = *Danio rerio*, Hs = *Homo sapiens* and Sc = *Saccharomyces cerevisiae*. (XLSX 57 kb)

Additional file 2: Es_seqannot.zip containing sequencing (fasta) files of the analyzed genes from *Echiniscoides* cf. *sigismundi*. (7Z 133 kb)

Additional file 3: Rc_seqannot.zip containing sequencing (fasta) files of the analyzed genes from *Richtersius* cf. *coronifer*. (7Z 177 kb)

## Data Availability

RNA-Seq raw data have been deposited in DDBJ/ENA/GenBank. Accession IDs for *Echiniscoides* cf. *sigismundi*: BioProject = PRJNA357357, BioSample = SAMN06141060, SRA = SRR7309271 and for *Richtersius* cf. *coronifer* BioProject = PRJNA475961, BioSample = SAMN09407000, SRA = SRR7340056. Transcript and protein predictions from the BGI assembly has been uploaded to UCPH ERDA at: 10.17894/ucph.9d3a898c-37bb-4cd1-909f-e7fcb07e58d9. The Es_CSP sequence is deposited in GenBank under the accession number MH500793.

## Abbreviations

53BP1: p53 binding protein 1
APTX: Aprataxin
ATHL: Acid trehalase
BER: Base excision repair
BRCA: Breast cancer
BUSCO: Benchmarking universal single-copy orthologs
CAHS: Cytosolic-abundant heat soluble
CAT: Catalase
CDK7: Cyclin dependent kinase 7
CEGMA: Core eukaryotic genes mapping approach
c-NHEJ: classical non-homologous end joining
COXI: Cytochrome c oxidase subunit I
CSD/ CSP: Cold shock domain/protein
CtIP: C-terminal-binding protein interacting protein
CuZn-SOD: CuZn superoxide dismutase
DSB: Double-strand break
Dur-1: Dauer upregulated-1
IIH: General transcription and DNA repair factor IIH
EME1: Essential meiotic structure-specific endonuclease 1
ERCC: Excision repair cross-complementation
Es: *Echiniscoides* cf. *sigismundi*
EXO1: Exonuclease 1
FEN1: Flap endonuclease 1
FPKM: Fragments per kilobase million
G6PD: Glucose-6-phosphate dehydrogenase
GPX: Glutathione peroxidase
GSS: Glutathione synthetase
GST: Glutathione S-transferase
HGT: Horizontal gene transfer
HR: Homologous recombination
HSP: Heat shock protein
HGNC: HUGO gene nomenclature committee
LEA: Late embryogenesis abundant
LIN-28: Abnormal cell lineage family member-28
MAHS: Mitochondrial abundant heat soluble
MLH: Mutator L (MutL) homolog
Mre11: Meiotic recombination protein 11
MSH: Mutator S (MutS) homolog
MUS81: MUS81 structure-specific endonuclease subunit
NBS1: Nibrin
NER: Nucleotide excision repair
p53/p63/p73: p53 superfamily involved in DNA repair
PARP: Poly (ADP-ribose) polymerase
PEX: Peroxin protein
PGGHG: Protein-glucosylgalactosylhydroxylysine glucosidase
POLB/POLE: DNA polymerase beta/epsilon
PRDX: Peroxiredoxin
PTS: Peroxisome targeting signal
PXMP/PMP: Peroxisomal membrane proteins
RAD50/RAD51: Radiation sensitive 50/ 51
RAxML: Randomized axelerated maximum likelihood
Rc: *Richtersius* cf. *coronifer*
RING: Really interesting new gene
ROS/RNS: Reactive oxygen/nitrogen species
Rv: *Ramazzottius* cf. *varieornatus*
RvLEAM: *Ramazzottius varieornatus* late embryogenesis abundant protein mitochondrial
SAHS: Secretory abundant heat soluble
SLX: Structure-specific endonuclease subunit
SOD: Superoxide dismutase
TPM: Transcripts per million
TPS: Trehalose-6-phosphate synthase
TREH: Trehalase
TXN: Thioredoxin
TXNRD: Thioredoxin reductase
TXNL: Thioredoxin like
TMX: Thioredoxin related transmembrane protein
TYSND1: Trypsin domain containing 1
XRCC: X-ray repair cross complementing
YB: Y-box

## References

[1] Møbjerg N, Halberg KA, Jørgensen A, Persson D, Bjørn M, Ramløv H, Kristensen RM (2011). Survival in extreme environments–on the current knowledge of adaptations in tardigrades. Acta Physiol.

[2] Nelson DR, Guidetti R, Tardigrada RLP, Thorp J, Rogers DC (2015). Ecology and general biology. Thorp and Covich's freshwater invertebrates volume 1.

[3] Jönsson KI, Rabbow E, Schill RO, Harms-Ringdahl M, Rettberg P (2008). Tardigrades survive exposure to space in low earth orbit. Curr Biol.

[4] Rebecchi L, Altiero T, Guidetti R, Cesari M, Bertolani R, Negroni M (2009). Tardigrade resistance to space effects: first results of experiments on the LIFE-TARSE mission on FOTON-M3 (September 2007). Astrobiology.

[5] Persson D, Halberg KA, Jørgensen A, Ricci C, Møbjerg N, Kristensen RM (2011). Extreme stress tolerance in tardigrades: surviving space conditions in low earth orbit. J Zool Syst Evol Res.

[6] Erdmann W, Kaczmarek Ł (2017). Tardigrades in space research - past and future. Orig Life Evol Biosph.

[7] Jørgensen A, Kristensen RM, Møbjerg N, Schill RO (2018). Phylogeny and integrative taxonomy of Tardigrada. Water bears: the biology of tardigrades. Series: zoological monographs, Vol. 2.

[8] Maas A, Braun A, Dong X-P, Donoghue PC, Müller KJ, Olempska E (2006). The ‘Orsten’—more than a Cambrian Konservat-Lagerstätte yielding exceptional preservation. Palaeoworld.

[9] Regier JC, Shultz JW, Kambic RE, Nelson DR (2004). Robust support for tardigrade clades and their ages from three protein-coding nuclear genes. Invertebr Biol.

[10] Sanders KL, Lee MS (2010). Arthropod molecular divergence times and the Cambrian origin of pentastomids. Syst Biodivers.

[11] Schokraie E, Hotz-Wagenblatt A, Warnken U, Mali B, Frohme M, Förster F (2010). Proteomic analysis of tardigrades: towards a better understanding of molecular mechanisms by anhydrobiotic organisms. PLoS One.

[12] Förster F, Beisser D, Grohme MA, Liang C, Mali B, Siegl AM (2012). Transcriptome analysis in tardigrade species reveals specific molecular pathways for stress adaptations. Bioinf Biol Insights.

[13] Boothby TC, Tenlen JR, Smith FW, Wang JR, Patanella KA, Nishimura EO (2015). Evidence for extensive horizontal gene transfer from the draft genome of a tardigrade. Proc Natl Acad Sci U S A.

[14] Koutsovoulos G, Kumar S, Laetsch DR, Stevens L, Daub J, Conlon C (2016). No evidence for extensive horizontal gene transfer in the genome of the tardigrade *Hypsibius dujardini*. Proc Natl Acad Sci U S A.

[15] Yamaguchi A, Tanaka S, Yamaguchi S, Kuwahara H, Takamura C, Imajoh-Ohmi S (2012). Two novel heat-soluble protein families abundantly expressed in an anhydrobiotic tardigrade. PLoS One.

[16] Hashimoto T, Horikawa DD, Saito Y, Kuwahara H, Kozuka-Hata H, Shin T (2016). Extremotolerant tardigrade genome and improved radiotolerance of human cultured cells by tardigrade-unique protein. Nat Commun.

[17] Yoshida Y, Koutsovoulos G, Laetsch DR, Stevens L, Kumar S, Horikawa DD (2017). Comparative genomics of the tardigrades *Hypsibius dujardini* and *Ramazzottius varieornatus*. PLoS Biol.

[18] Hygum TL, Clausen LK, Halberg KA, Jørgensen A, Møbjerg N (2016). Tun formation is not a prerequisite for desiccation tolerance in the marine tidal tardigrade *Echiniscoides sigismundi*. Zool J Linnean Soc.

[19] Møbjerg N, Kristensen RM, Jørgensen A (2016). Data from new taxa infer *Isoechiniscoides* gen. Nov. and increase the phylogenetic and evolutionary understanding of echiniscoidid tardigrades (Echiniscoidea: Tardigrada). Zool J Linnean Soc.

[20] Gąsiorek P, Stec D, Morek W, Michalczyk Ł (2018). An integrative redescription of *Hypsibius dujardini* (Doyère, 1840), the nominal taxon for Hypsibioidea (Tardigrada: Eutardigrada). Zootaxa.

[21] Morek W, Gąsiorek P, Stec D, Blagden B, Michalczyk Ł (2016). Experimental taxonomy exposes ontogenetic variability and elucidates the taxonomic value of claw configuration in *Milnesium* Doyère, 1840 (Tardigrada: Eutardigrada: Apochela). Contrib Zool.

[22] Erkut C, Vasilj A, Boland S, Habermann B, Shevchenko A, Kurzchalia TV (2013). Molecular strategies of the *Caenorhabditis elegans* dauer larva to survive extreme desiccation. PLoS One.

[23] Tanaka S, Tanaka J, Miwa Y, Horikawa DD, Katayama T, Arakawa K (2015). Novel mitochondria-targeted heat-soluble proteins identified in the anhydrobiotic tardigrade improve osmotic tolerance of human cells. PLoS One.

[24] Stamatakis A (2014). RAxML version 8: a tool for phylogenetic analysis and post-analysis of large phylogenies. Bioinformatics.

[25] Guindon S, Dufayard JF, Lefort V, Anisimova M, Hordijk W, Gascuel O (2010). New algorithms and methods to estimate maximum-likelihood phylogenies: assessing the performance of PhyML 3.0. Syst Biol.

[26] Mali B, Grohme MA, Förster F, Dandekar T, Schnölzer M, Reuter D (2010). Transcriptome survey of the anhydrobiotic tardigrade *Milnesium tardigradum* in comparison with *Hypsibius dujardini* and *Richtersius coronifer*. BMC Genomics.

[27] Smith FW, Boothby TC, Giovannini I, Rebecchi L, Jockusch EL, Goldstein B (2016). The compact body plan of tardigrades evolved by the loss of a large body region. Curr Biol.

[28] Wright JC (1989). Desiccation tolerance and water-retentive mechanisms in tardigrades. J Exp Biol.

[29] Clegg JS (2001). Cryptobiosis—a peculiar state of biological organization. Comp Biochem Physiol B.

[30] Madin K, Crowe JH (1975). Anhydrobiosis in nematodes: carbohydrate and lipid metabolism during dehydration. J Exp Zool A.

[31] Clegg JS (1962). Free glycerol in dormant cysts of the brine shrimp *Artemia salina*, and its disappearance during development. Biol Bull.

[32] Watanabe M, Kikawada T, Okuda T (2003). Increase of internal ion concentration triggers trehalose synthesis associated with cryptobiosis in larvae of *Polypedilum vanderplanki*. J Exp Biol.

[33] Lapinski J, Tunnacliffe A (2003). Anhydrobiosis without trehalose in bdelloid rotifers. FEBS Lett.

[34] Westh P, Ramløv H (1991). Trehalose accumulation in the tardigrade *Adorybiotus coronifer* during anhydrobiosis. J Exp Zool A.

[35] Jönsson KI, Persson O (2010). Trehalose in three species of desiccation tolerant tardigrades. Open Zool J.

[36] Hengherr S, Heyer AG, Köhler HR, Schill RO (2008). Trehalose and anhydrobiosis in tardigrades – evidence for divergence in responses to dehydration. FEBS J.

[37] Battaglia M, Olvera-Carrillo Y, Garciarrubio A, Campos F, Covarrubias AA (2008). The enigmatic LEA proteins and other hydrophilins. Plant Physiol.

[38] Tunnacliffe A, Hincha DK, Leprince O, Macherel D, Lubzens E, Cerda J, Clark M (2010). LEA proteins: versatility of form and function. Dormancy and resistance in harsh environments.

[39] Boschetti C, Pouchkina-Stantcheva N, Hoffmann P, Tunnacliffe A (2011). Foreign genes and novel hydrophilic protein genes participate in the desiccation response of the bdelloid rotifer *Adineta ricciae*. J Exp Biol.

[40] Chakrabortee S, Tripathi R, Watson M, Kaminski Schierle GS, Kurniawan DP (2012). Intrinsically disordered proteins as molecular shields. Mol BioSyst.

[41] Goyal K, Walton LJ, Browne JA, Burnell AM, Tunnacliffe A (2005). Molecular anhydrobiology: identifying molecules implicated in invertebrate anhydrobiosis. Integr Comp Biol.

[42] Hand SC, Menze MA, Toner M, Boswell L, Moore D (2011). LEA proteins during water stress: not just for plants anymore. Annu Rev Physiol.

[43] Boothby TC, Tapia H, Brozena AH, Piszkiewicz S, Smith AE, Giovannini I (2017). Tardigrades use intrinsically disordered proteins to survive desiccation. Mol Cell.

[44] Clausen LKB, Andersen KN, Hygum TL, Jørgensen A, Møbjerg N (2014). First record of cysts in the tidal tardigrade *Echiniscoides sigismundi*. Helgol Mar Res.

[45] Sørensen-Hygum TL, Stuart RM, Jørgensen A, Møbjerg N (2018). Modelling extreme desiccation tolerance in a marine tardigrade. Sci Rep.

[46] Ellis RJ (2001). Molecular chaperones: inside and outside the Anfinsen cage. Curr Biol.

[47] Mu H, Sun J, Fang L, Luan T, Williams GA, Cheung SG (2015). Genetic basis of differential heat resistance between two species of congeneric freshwater snails: insights from quantitative proteomics and base substitution rate analysis. J Proteome Res.

[48] Tantos A, Friedrich P, Tompa P (2009). Cold stability of intrinsically disordered proteins. FEBS Lett.

[49] Beauchemin M, Roy S, Pelletier S, Averback A, Lanthier F, Morse D (2016). Characterization of two dinoflagellate cold shock domain proteins. mSphere.

[50] Graumann PL, Marahiel MA (1998). A superfamily of proteins that contain the cold-shock domain. Trends Biochem Sci.

[51] Foyer CH, Rasool B, Davey JW, Hancock RD (2016). Cross-tolerance to biotic and abiotic stresses in plants: a focus on resistance to aphid infestation. J Exp Bot.

[52] Adler Victor, Yin Zhimin, Tew Kenneth D, Ronai Ze'ev (1999). Role of redox potential and reactive oxygen species in stress signaling. Oncogene.

[53] Kültz D (2005). Molecular and evolutionary basis of the cellular stress response. Annu Rev Physiol.

[54] Bonekamp NA, Völkl A, Fahimi HD, Schrader M (2009). Reactive oxygen species and peroxisomes: struggling for balance. Biofactors.

[55] Wełnicz W, Grohme MA, Kaczmarek Ł, Schill RO, Frohme M (2011). Anhydrobiosis in tardigrades—the last decade. J Insect Physiol.

[56] Rizzo AM, Negroni M, Altiero T, Montorfano G, Corsetto P, Berselli P (2010). Antioxidant defences in hydrated and desiccated states of the tardigrade *Paramacrobiotus richtersi*. Comp Biochem Physiol B.

[57] Hygum TL, Fobian D, Kamilari M, Jørgensen A, Schiøtt M, Grosell M, et al. Comparative investigation of copper tolerance and identification of putative tolerance related genes in tardigrades. Front Physiol. 2017;8:95. 10.3389/fphys.2017.00095. 10.3389/fphys.2017.00095 PMC5328964 28293195

[58] Beltrán-Pardo E, Jönsson KI, Harms-Ringdahl M, Haghdoost S, Wojcik A (2015). Tolerance to gamma radiation in the tardigrade *Hypsibius dujardini* from embryo to adult correlate inversely with cellular proliferation. PLoS One.

[59] Fransen M, Nordgren M, Wang B, Apanasets O (2012). Role of peroxisomes in ROS/RNS-metabolism: implications for human disease. Biochim Biophys Acta (BBA) - Mol Basis Dis.

[60] Zamocky M, Furtmüller PG, Obinger C (2008). Evolution of catalases from bacteria to humans. Antioxid Redox Signal.

[61] Ishikawa T, Tamaki S, Maruta T, Shigeoka S, Schwartzbach S, Shigeoka S (2017). Biochemistry and physiology of reactive oxygen species in *Euglena*. Euglena: biochemistry, cell and molecular biology.

[62] Ho Y-S, Xiong Y, Ma W, Spector A, Ho DS (2004). Mice lacking catalase develop normally but show differential sensitivity to oxidant tissue injury. J Biol Chem.

[63] Joanisse DR, Storey KB (1998). Oxidative stress and antioxidants in stress and recovery of cold-hardy insects. Insect Biochem Mol Biol.

[64] Ma C, Agrawal G, Subramani S (2011). Peroxisome assembly: matrix and membrane protein biogenesis. J Cell Biol.

[65] Smith JJ, Aitchison JD (2013). Peroxisomes take shape. Nat Rev Mol Cell Biol.

[66] Van Veldhoven PP, Baes M. Peroxisome deficient invertebrate and vertebrate animal models. Front Physiol. 2013;4:335. 10.3389/fphys.2013.00335. 10.3389/fphys.2013.00335 PMC3837297 24319432

[67] Baker A, Carrier DJ, Schaedler T, Waterham HR, van Roermund CW, Theodoulou FL (2015). Peroxisomal ABC transporters: functions and mechanism. Biochem Soc Trans.

[68] Neumann S, Reuner A, Brümmer F, Schill RO (2009). DNA damage in storage cells of anhydrobiotic tardigrades. Comp Biochem Physiol A.

[69] Horikawa DD, Cumbers J, Sakakibara I, Rogoff D, Leuko S, Harnoto R (2013). Analysis of DNA repair and protection in the tardigrade *Ramazzottius varieornatus* and *Hypsibius dujardini* after exposure to UVC radiation. PLoS One.

[70] Rizzo AM, Altiero T, Corsetto PA, Montorfano G, Guidetti R, Rebecchi L (2015). Space flight effects on antioxidant molecules in dry tardigrades: the TARDIKISS experiment. Biomed Res Int.

[71] Dexheimer TS (2013). DNA repair pathways and mechanisms. DNA repair of cancer stem cells.

[72] Bertolani R (2001). Evolution of the reproductive mechanisms in tardigrades—a review. Zool Anz.

[73] Czernekova M, Jönsson KI (2016). Mitosis in storage cells of the eutardigrade *Richtersius coronifer*. Zool J Linnean Soc.

[74] Deng W, Henriet S, Chourrout D (2018). Prevalence of mutation-prone microhomology-mediated end joining in a chordate lacking the c-NHEJ DNA repair pathway. Curr Biol.

[75] Wei L, Levine AS, Lan L (2016). Transcription-coupled homologous recombination after oxidative damage. DNA Repair.

[76] Halberg KA, Jørgensen A, Møbjerg N (2013). Desiccation tolerance in the tardigrade *Richtersius coronifer* relies on muscle mediated structural reorganization. PLoS One.

[77] Jørgensen A, Møbjerg N, Kristensen RM (2011). Phylogeny and evolution of the Echiniscidae (Echiniscoidea, Tardigrada)–an investigation of the congruence between molecules and morphology. J Zool Syst Evol Res.

[78] Grabherr MG, Haas BJ, Yassour M, Levin JZ, Thompson DA, Amit I (2011). Full-length transcriptome assembly from RNA-Seq data without a reference genome. Nat Biotechnol.

[79] Mortazavi A, Williams BA, McCue K, Schaeffer L, Wold B (2008). Mapping and quantifying mammalian transcriptomes by RNA-Seq. Nat Methods.

[80] Trapnell C, Williams BA, Pertea G, Mortazavi A, Kwan G, Van Baren MJ (2010). Transcript assembly and quantification by RNA-Seq reveals unannotated transcripts and isoform switching during cell differentiation. Nat Biotechnol.

[81] Wagner GP, Kin K, Lynch VJ (2012). Measurement of mRNA abundance using RNA-seq data: RPKM measure is inconsistent among sample. Theory Biosci.

[82] Conesa A, Götz S, García-Gómez JM, Terol J, Talón M, Robles M (2005). Blast2GO: a universal tool for annotation, visualization and analysis in functional genomics research. Bioinformatics.

[83] Parra G, Bradnam K, Korf I (2007). CEGMA: a pipeline to accurately annotate core genes in eukaryotic genomes. Bioinformatics.

[84] Simão FA, Waterhouse RM, Ioannidis P, Kriventseva EV, Zdobnov EM (2015). BUSCO: assessing genome assembly and annotation completeness with single-copy orthologs. Bioinformatics.

[85] Gramates LS, Marygold SJ, Gd S, Urbano J-M, Antonazzo G, Matthews BB (2017). FlyBase at 25: looking to the future. Nucleic Acids Res.

[86] James-Zorn C, Ponferrada VG, Burns KA, Fortriede JD, Lotay VS, Liu Y (2015). Xenbase: Core features, data acquisition, and data processing. Genesis.

[87] Karpinka JB, Fortriede JD, Burns KA, James-Zorn C, Ponferrada VG, Lee J (2015). Xenbase, the *Xenopus* model organism database; new virtualized system, data types and genomes. Nucleic Acids Res.

[88] Howe K, Clark MD, Torroja CF, Torrance J, Berthelot C, Muffato M (2013). The zebrafish reference genome sequence and its relationship to the human genome. Nature.

[89] Cherry JM, Hong EL, Amundsen C, Balakrishnan R, Binkley G, Chan ET (2011). *Saccharomyces* genome database: the genomics resource of budding yeast. Nucleic Acids Res.

[90] Li L, Stoeckert CJ, Roos DS (2003). OrthoMCL: identification of ortholog groups for eukaryotic genomes. Genome Res.

[91] Chen F, Mackey AJ, Stoeckert CJ, Roos DS (2006). OrthoMCL-DB: querying a comprehensive multi-species collection of ortholog groups. Nucleic Acids Res.

[92] Stöver BC, Müller KF (2010). TreeGraph 2: combining and visualizing evidence from different phylogenetic analyses. BMC Bioinf.

